# The Changes in Bacterial Microbiome Associated with Immune Disorder in Allergic Respiratory Disease

**DOI:** 10.3390/microorganisms10102066

**Published:** 2022-10-19

**Authors:** Juanjuan Lyu, Fangfang Kou, Xiangyu Men, Yinhui Liu, Li Tang, Shu Wen

**Affiliations:** Department of Microecology, College of Basic Medical Sciences, Dalian Medical University, Dalian 116044, China

**Keywords:** allergic rhinitis, asthma, chronic rhinosinusitis, immune disorder, bacterial microbiome

## Abstract

Allergic respiratory disease is a worldwide and increasingly prevalent health problem. Many researchers have identified complex changes in the microbiota of the respiratory and intestinal tracts in patients with allergic respiratory diseases. These affect immune response and influence the progression of disease. However, the diversity of bacterial changes in such cases make it difficult to identify a specific microorganism to target for adjustment. Recent research evidence suggests that common bacterial variations present in allergic respiratory disease are associated with immune disorders. This finding could lead to the discovery of potential therapeutic targets in cases of allergic respiratory disease. In this review, we summarize current knowledge of bacteria changes in cases of allergic respiratory disease, to identify changes commonly associated with immune disorders, and thus provide a theoretical basis for targeting therapies of allergic respiratory disease through effective modulation of key bacteria.

## 1. Introduction

A growing body of evidence reveals an increased prevalence of allergic respiratory diseases in developing countries. Allergic respiratory diseases include allergic rhinitis (AR), chronic rhinosinusitis (CRS) with nasal polyps, and asthma, all of which affect work, physical health, quality of life, etc., and impose a serious economic burden on individuals and societies. These diseases, with a group of immune disorders, are characterized by immune inflammation with elevated levels of immunoglobulin (Ig) E and are usually associated with over-activation of type 2 immune response. T helper type 9 (Th9) lymphocytes and Th17 cells are involved in the immune response. Researchers have found that bacterial microbiota dysbiosis is commonly present in the development of allergic respiratory diseases [1,2,3]. The commensal microbiota plays an important role in host immune co-ordination, and help to maintain the integrity of the mucosal and epithelial barrier [4]. Recent findings suggest that bacterial changes are associated with mucosal inflammation, especially increased levels of bacteria, such as *Moraxella* and *Haemophilus*, which may promote immune imbalances and increase the severity of disease [1]. For this reason, regulation of potential “key genera” might form the basis of new therapeutic strategy. For any given disease, the unpredictability of associated bacterial changes due to multiple factors makes it difficult to determine those bacteria with value as therapeutic targets. However, because commonly bacterial changes present in patients with allergic respiratory diseases are associated with immune disorders, these changes may represent potential therapeutic targets for treatment of such diseases. In this review, we summarize current knowledge concerning changes in bacterial microbiota associated with allergic respiratory diseases, to identify bacterial changes commonly associated with immune disorders, and thus provide a theoretical basis for finding therapeutic targets for allergic respiratory diseases in the future.

## 2. Immune Mechanism of Allergic Respiratory Diseases

**Figure 1:**
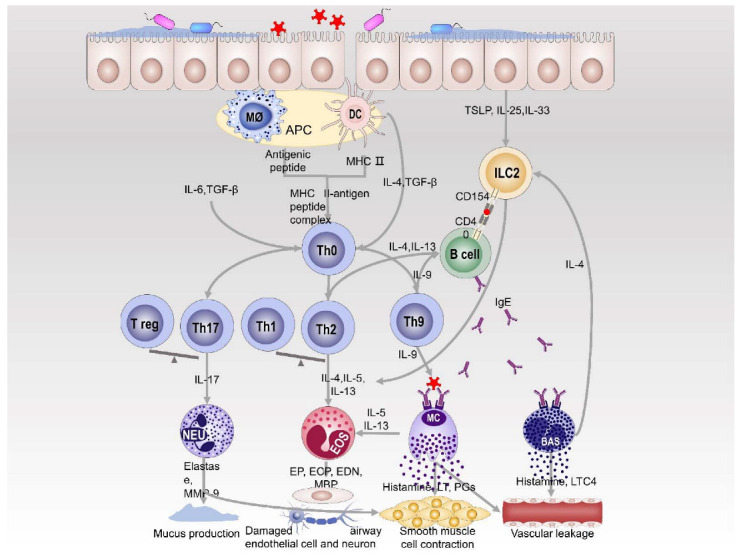
Immune mechanism of allergic respiratory diseases. Allergens are recognized and processed by antigen-presenting cells to form major histocompatibility complex (MHC) class II molecules-antigen peptide complexes that promote the differentiation of T helper (Th) cells into Th2 cells, which product pro-inflammatory cytokines (IL-4, IL-5, IL-13) to activate eosinophils and stimulate IgE synthesis by B cells. Th0 cells are stimulated by different cytokines to differentiate into Th9, Th17 cells and promote the production of IL-9, IL-17. These cytokines activate the corresponding effector cells to produce a variety of active substances leading to immune inflammation. In addition, epithelial cells stimulated by allergens secrete IL-25, IL-33 and thymic stromal lymphopoietin (TSLP) to activate group 2 innate lymphoid cells (ILC2s) that secrete various pro-inflammatory cytokines to act on the corresponding effector cells. Abbreviations: DC, dendritic cells; Mø, macrophages; NEU, neutrophils; EOS, eosinophils; MC, mast cells; BAS, basophils; MMP-9, matrix metalloproteinase-9; EP, eosinophil peroxidase; ECP, eosinophil cationic protein; EDN, eosinophilic neurotoxin; MBP, major basic protein; LT, leukotrienes; PGs, prostaglandins.

Allergic respiratory diseases are characterized by enhanced levels of Th2 immune response, elevated levels of IgE, and increase in eosinophilia. In the development of allergic respiratory diseases, Th9 cells, Th17cells, group 2 innate lymphoid cells (ILC2s), and mast cells also play significant roles [5,6,7]. Allergens affect the differentiation of Th0 cells to Th2, Th9, and Th17 cells through antigen presenting cells (APCs), promoting the production of pro-inflammatory cytokines and IgE [8,9,10,11,12,13]. Some recently identified ILC2s are also involved in this process. The above products activate effector cells to release inflammatory mediators that cause immune inflammation in the airway [14]. The immune responses’ cascades ultimately lead to the development of multiple clinical symptoms of allergic respiratory diseases (Figure 1). 

Immune disorders in allergic respiratory diseases involve multiple pathways, including immune imbalance of Th1/Th2 and Treg/Th17, and the activation of a range of immune cells. Immune disturbances in allergic respiratory diseases are influenced by a variety of factors that include antibiotic use and bacterial exposure (e.g., *Moraxella catarrhala* and *Haemophilus influenzae*) [15,16]. With the development of next-generation gene sequencing technology, researchers have become more aware of bacterial changes at different sites. This is an area of study that should be further explored.

## 3. Characteristics of Bacterial Microbiome Changes in the Respiratory tract and Intestine

The upper airway is now considered to be the main entry point for pathogens and a reservoir for respiratory symbiotic microbiota. For many years, the lungs were considered a sterile environment; with the development of next-generation sequencing, colonization of bacteria in the lungs has been confirmed. A growing number of studies into the relationship between allergic respiratory diseases (AR, CRS, asthma) and bacteria have found substantial bacterial changes at different sites in patients’ bodies. Typically, these changes are complex and difficult to categorize. However, some researchers have noted the association between immune responses and common bacterial changes in cases of allergic respiratory diseases. In this article, we summarize recently published studies of bacterial variation in allergic respiratory diseases to identify commonly changed bacteria. We note the different bacterial sample distribution sites associated with different allergic respiratory diseases in recent studies. In the current study, samples from AR patients analyzed were mainly collected from the upper respiratory tract and intestine, samples from CRS patients were mainly focused on the upper respiratory tract, and samples from asthmatic patients were more widely distributed, including upper and lower respiratory tract and intestinal tract.

### 3.1. Bacteria Characteristics of Allergic Respiratory Diseases in the Upper Respiratory Tract and Intestine

#### 3.1.1. Changes in Upper Respiratory Tract Bacteria in Allergic Respiratory Diseases

Many researchers have pointed out that upper airway microbiota dysbiosis seriously affects the health [2,3,17,18,19,20,21,22,23,24,25,26,27,28,29,30,31,32,33,34,35,36,37,38,39,40,41,42,43,44,45,46,47,48]. Published reports confirm the presence of bacterial disorder in the upper respiratory tract of patients with AR, CRS, and asthma (Figures S1A and S2). Researchers have found increases in bacteria of the phylum Proteobacteria, [1,3,17,18] with large increases in the class Gammaproteobacteria. Among these, *Moraxella* [3,17,29,31,34,35,36,37] increases in cases of all three diseases, while *Haemophilus* [2,28,34,35,39,48] levels increase in the upper airway of patients with AR and CRS. *Pseudomonas* increases in the upper respiratory tract of AR patients, and although it declines in CRS patients, two species belonging to *Pseudomonas* have been found to increase in the same populations [26,27,32]. In addition, the prevalence of *Neisseria* (Betaproteobacteria) also rises in AR and CRS [28,34]. Most of these genera are positively associated with inflammation or pro-inflammatory cytokines, suggesting that the phylum Proteobacteria, especially those genera belonging to Gammaproteobacteria, may be associated with the development of allergic respiratory diseases.

Turning to other resident commensal bacteria that predominate in the nasal cavity, researchers have found complex changes in cases of all three allergic respiratory diseases. Considerably lower levels of Bacteroidetes are present in the upper respiratory tracts of AR and CRS patients [19,20], but higher levels are exhibited in asthmatic patients [1]. *Prevotella* is a representative of this trend in the phylum Bacteroidetes [1,2,20,21,31,32,39,41], while complex changes have also been found in genera of the phylum Firmicutes. Levels of *Staphylococcus* rise in both CRS and asthma patients [20,21,27,39,44], though the prevalence of this genus decreases in the upper airway of AR patients [17] and the levels of *Staphylococcus aureus* increase [19,25,29,32,38,47]. Furthermore, the prevalence of *Streptococcus* in the upper respiratory tract is higher in both CRS and asthma patients [28,29,36,43], while levels of *Butyrivibro* decline in AR and asthma patients [34]. Moreover, the abundance of Actinobacteria is significantly reduced in AR and CRS patients, despite variations in results for particular genera exhibited across the three diseases [17,18,22,23,24].

When we analyze changes in bacterial microbiota at different levels, we find relatively consistent changes in *Moraxella*, *Haemophilus*, *Neisseria*, *Prevotella*, *Pseudomonas*, *Streptococcus,* and *Staphylococcus aureus* in the upper airway of patients with allergic respiratory diseases, indicating possible association between bacterial changes and the development of allergic respiratory diseases. The impact of host–microbe interactions extends well beyond the local environment, thereby influencing the response of peripheral tissues. We know that important crosstalk exists between mucosal tissues in the human body, as in the gut–lung axis, although the mechanisms of interactions between the pulmonary and intestinal environments are not yet clear.

#### 3.1.2. Changes of Intestinal Bacteria in Allergic Respiratory Diseases

A pooled analysis of multiple studies confirms that there are various bacterial changes in the gut of patients with allergic respiratory disease [49,50,51,52,53,54,55,56,57,58,59,60,61,62,63]. For this review, we mainly considered changes in gut bacteria in patients with AR and asthma, as only a few 16srRNA gene sequence analyses have been performed on gut bacteria of patients with CRS (Figures S1B and S2), and these findings in CRS show increased levels of *Enterobacter* and a decline in *Bifidobacterium* [31]. 

Then, turning to the signature of gut bacteria in patients with AR and asthma, we find that the genera *Escherichia-Shigella*, *Escherichia* (Gammaproteobacteria), and *Bilophila* (Deltaproteobacteria) increased in both diseases [41,50,51,52,55,60], while the genus in Bacteroides that most commonly declines is *Alistipes* [49,52]. The prevalence of *Prevotella* (Bacteroides) is substantially lower in the intestinal tracts of AR patients, but higher in asthmatic patients, with corresponding changes in the upper airway. Changes in taxa below the phylum Firmicutes are typically more complex in AR and asthma patients, though levels of most genera and families belonging to Bacilli were increased. Genera belonging to Clostridia and Negativicutes exhibit complex changes; for example, levels of *Clostridium* and *Phascolarctobacterium* increase, while the prevalence of *Faecalibacterium* and *Dialister* declines [41,49,50,51,52,55,61,62] in AR and asthma. Changes in taxonomic classification below the phylum Actinobacteria are more difficult to assess. For example, in one study of the intestines of patients with AR, levels of Actinobacteria and Bifidobacteriaceae declined while the prevalence of *Bifidobacterium* increased [49]. Other researchers found reduced levels of *Bifidobacterium*, while one study produced a contrary result [52,54,56,60]. In addition, *Adlercreutzia* and *Eggerthella* of the Eggerthellaceae family have been found at high levels in patients with AR and asthma, respectively [51,52,55]. Furthermore, increased levels of the genera and family from Fusobacteria have been reported in both diseases [49,57]. Overall, when we consider changes in gut bacterial composition in patients with AR and asthma, we find a complex and inconsistent picture. 

### 3.2. Changes in the Bacteria of the Lower Respiratory Tract in Asthma

**Figure 2:**
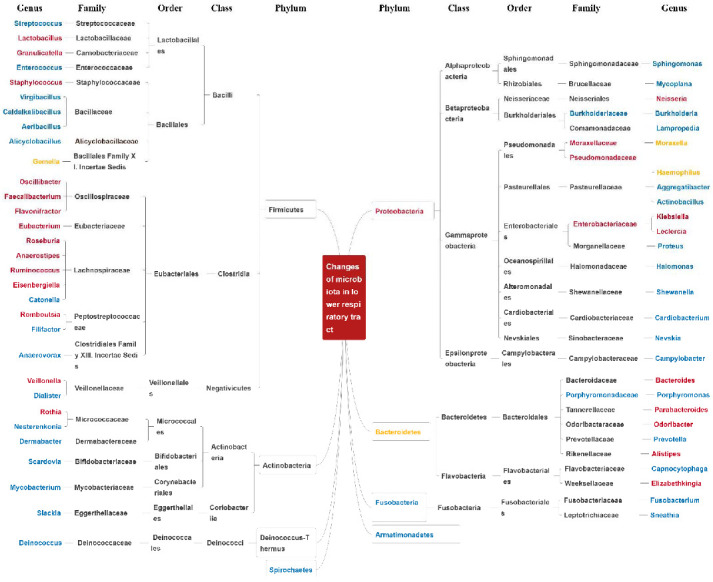
Changes in the bacteria of the lower respiratory tract in asthma. Red represents increased prevalence, blue represents decreased prevalence, and yellow represents uncertain changes in prevalence.

Recent studies of bacterial microbiome changes in the lower respiratory tract in cases of allergic respiratory diseases have been mainly restricted to asthmatic patients [64,65,66,67,68,69,70,71,72,73,74,75]. Researchers have found such changes to be complex and diverse, but some common characteristics have been identified (Figure 2). In asthmatic patients, a higher relative abundance of Proteobacteria [64,65,66] is an obvious signature in the lower airway, just as in the upper airway. At the genus level, an increased prevalence of *Moraxella* [66,71,72,73,75] or *Staphylococcus* [65] in the lower respiratory tract is consistent with similar changes in the upper respiratory tract. Moreover, an increase in *Haemophilus* [65,71,72,75] and *Neisseria* [70] and a decrease in *Prevotella* [65] in the lower respiratory tract of asthmatic patients is compatible with changes in these bacteria in the upper airway in cases of the other allergic diseases, indicating a possible correlation between these changes and allergic inflammation. However, many bacterial changes in the lower respiratory tract differ from those exhibited in the upper respiratory tract, including increases in *Bacteroides* and *Parabacteroides* [67,70] and a decrease in *Porphyromonas* [71,75]. Further studies into these complicated and diverse bacterial changes are required. 

Although bacteria changes at different sites in patients with allergic respiratory diseases are generally complex and unpredictable, we can identify some common signatures. For instance, *Moraxella*, *Haemophilus*, *Neisseria*, *Pseudomonas*, *Staphylococcus aureus,* and *Streptococcus*, all of which are universally present in the airway, are typically increased in cases of allergic respiratory disease, while the gut bacteria *Escherichia*, *Clostridium*, *Bilophila*, *Phascolarctobacterium*, *Dialister*, *Bifidobacterium*, *Faecalibacterium*, *Lactobacillus,* and *Prevotella* frequently exhibit similar changes in the gut in AR and asthma, with different change results among these bacteria. Some bacteria such as *Prevotella* also exhibit changes in the intestine and the airways. These above-mentioned genera whose prevalence most commonly changes are those most likely to be responsible for allergic respiratory diseases.

Eighteen of the aforementioned papers that assessed changes in the bacterial microbiota involved studies of infants and children [3,17,21,36,37,41,42,43,51,54,55,56,59,60,61,62,67,73], whose bacterial changes are generally consistent with those of adults. Birth cohort studies of infants have found that bacteria dysbiosis occurring early in life is strongly associated with the onset of allergic rhinitis and wheezing later in life [17,42,54]. This suggests that early bacterial changes have an impact on the development of allergic respiratory disease. These disease-associated bacteria are related to unique and localized airway immune features that further influence the development of allergic diseases.

For the purposes of this review, we note a lack of uniformity in previous studies, especially concerning the upper respiratory tract, i.e., the sinuses, anterior nose, nasopharynx, pharynx, and middle nasal tract. In studies to date, the sampling sites of patients with CRSwNP have been mainly concentrated in the middle nasal tract, but researchers investigating patients with asthma and AR have used a wide range of sampling sites in the upper respiratory tract, with possible consequent bias in results. Given the relatively short history of research in this area and the limited samples and data available, we had to use all available data in our analysis. The quality of the evidence was compromised by uneven and inconsistent sampling loci. More uniform evidence is required, and better-designed sampling norms in future studies will help to achieve this. However, we can see that changes in bacterial microbiota are similar in disease states, despite minor biases in sampling loci. It is possible that inflammation-induced environmental convergence leads to similar changes in microbiota. the exact cause of which should be further investigated and explored in future studies.

## 4. The Association between Changed Bacteria and Immune Disorders

**Figure 3:**
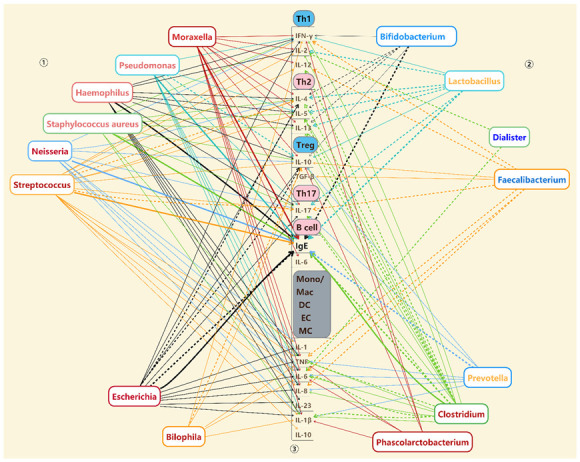
The relationship between co-changing bacteria and immune disturbances. ① Bacteria that promote immune inflammation and are positively correlated with IgE. ② Bacteria with anti-inflammatory effect. ③ Bacteria with some positive correlation with immune inflammation, but whose relationship with IgE is unclear. Emphasized relationships are in bold. Solid lines represent positive correlations and dashed lines represent negative correlations. Red words represent the increased prevalence of bacteria in at least two diseases at the same site, pink words represent the predominantly increased prevalence of the bacteria in two diseases at the same site, blue words represent the decreased prevalence of the bacteria in at least two diseases at the same site, light blue words represent the predominantly decreased prevalence of bacteria in two diseases at the same site, and yellow words represent the change in the same bacteria in different diseases which cannot be determined. Mono/Mac: monocytes/macrophages. MC: mast cells, EC: epithelial cells, DC: dendritic cells.

The extent and diversity of bacterial changes in allergic respiratory diseases make it difficult to identify specific microorganisms as targets for adjustment. However, we can note that allergic respiratory diseases are a group of immune dysregulation diseases associated with allergic immune imbalance (Th1/Th2, Treg/Th17 imbalance) as well as the accumulation of IgE. Current evidence indicates that these common bacterial changes in cases of allergic respiratory diseases are associated with these immune disorders; these affected bacteria might therefore serve as potential therapeutic targets for allergic respiratory diseases [76,77,78]. The relationship between covariant bacteria and immune inflammation is summarized in Figure 3, which shows that the commonly changed bacteria can be divided into three categories according to their relationship with immune inflammation, as follows: ① bacteria that promote immune inflammation and can increase IgE production; ② bacteria with anti-inflammatory effects; and ③ bacteria positively correlated with immune inflammation, but whose relationship with IgE is unclear.

The first category of bacteria includes six genera which promote the production of Th2 cytokines (IL-4, IL-5, IL-13), Th17 cytokines (IL-17), and other pro-inflammatory cytokines, and exhibit positively correlation with levels of IgE. Most of these bacteria are present in the respiratory tract and their prevalence increases in cases of allergic respiratory diseases. However, these bacteria exhibit differing degrees of pro-inflammatory and anti-inflammatory effects. Four of them, namely, *Moraxella*, *Staphylococcus aureus*, *Haemophilus,* and *Pseudomonas*, seem to trigger Th1/Th2 and Treg/Th17 imbalance, as well as release of IgE, although they have been positively correlated with anti-inflammatory cytokines [76,77,78,79,80,81,82,83,84,85,86,87,88,89,90,91,92,93,94]. The remaining genera, including *Neisseria* and *Streptococcus*, exhibit a positive correlation with IgE levels, but their connection with other immune cells is ambiguous [95,96,97,98,99,100,101,102,103,104,105,106,107,108,109].

The second category of bacteria which are mainly found in the intestine is characterized by increased production of anti-inflammatory cytokines and decreased production of pro-inflammatory cytokines, which have the capability to modulate immune dysregulation and build a defensive line against inflammation. Researchers have found that *Bifidobacterium* and *Lactobacillus* prevent Th1/Th2 and Treg/Th17 imbalance, as well as production of IgE [110,111,112,113,114]. Other studies have reported a negative correlation of *Dialister* and inflammatory cytokine production [115,116], as well as the action of *Faecalibacterium* as an inhibitor of inflammation, which mainly regulates Treg/Th17 imbalance [117,118,119]. Decreased levels of bacteria in this category destroy the line of defense against inflammation, which then increases uncontrollably, leading to favorable conditions for disease development.

The third group of bacteria is associated with pro-inflammatory cytokines secreted by natural immune cells, but any correlation between these and IgE production has not yet been determined. Typically, these bacteria exhibit changes in the intestinal tract of patients with allergic respiratory diseases. *Bilophila* and *Phascolarctobacterium* promote pro-inflammatory cytokines associated with innate immune cells while maintaining Th1/Th2 and Treg/Th17 balance to some extent [120,121,122,123,124,125]. Other bacteria in this group display a more complex and indeterminate association with inflammation [51,126,127,128,129,130,131,132,133,134,135,136]. *Escherichia* is [128,129] uncertainly associated with IgE production while *Prevotella* mainly promotes inflammation, but exhibits a negative association with production of IgE [131,132]. *Clostridium* exhibits both anti-inflammatory and pro-inflammatory effects [51,133,134,135,136] depending on the species concerned, so that *Clostridium butyricum* is negatively associated with inflammation, but *Clostridium difficile* exhibits an opposite effect, making the relationship between these bacteria and immune inflammation unclear.

Immune disorders associated with allergic respiratory diseases manifest themselves in an imbalance of Th1/Th2 and Treg/Th17, as well as increased production of IgE. To summarize, we note that the first category of bacteria (*Staphylococcus aureus*, *Haemophilus*, *Streptococcus*, *Pseudomonas*, *Moraxella*, etc.), which typically increases in the respiratory tract in allergic respiratory disease, is associated with an allergic immune inflammatory response, while such a response is inhibited by second category bacteria (*Bifidobacterium*, *Lactobacillus*, *Faecalibacterium*, etc.), which commonly decrease in the intestinal tract. Finally, the third category of bacteria, which increase in the intestinal tract, appear to promote common inflammation. The increase in pro-inflammatory bacteria and the decrease in anti-inflammatory bacteria promote the immune imbalance of Th1/Th2 and Treg/Th17. As the immune balance is disturbed, the associated allergic inflammation follows.

The above studies demonstrate that bacteria changes in allergic diseases are immune-related. It is worth noting that the timing of bacteria changes is important. During the first year of life, the developing bacterial community is critical for the maturation of immune function. Due to low antigen exposure in utero, the acquired immune system is naïve at birth, and its maturation occurs in the early postnatal period and is impacted by the infant’s diet and environment. At birth, the immune system is characterized by a predominant Th2 cytokine response, and maturation in infancy is associated with an improved Th1 response. The maintenance of an exaggerated Th2 response increases the risk of allergy and other atopic diseases. A prospective study of childhood asthma demonstrated that neonates colonized with *Moraxella catarrhalis*, *Haemophilus influenzae*, and *Streptococcus pneumoniae* had higher levels of congenital TH2/TH17-related cytokines and chemokines in the lining fluid of their upper airways compared with non-colonized neonates. Infants with pro-inflammatory responses caused by these bacteria were subsequently shown to be at higher risk of developing asthma at seven years of age. These findings suggest that early colonization by these pro-inflammatory bacteria may influence type 2 chronic inflammation and subsequent asthma development.

## 5. Treatment

The diverse bacterial changes displayed in allergic respiratory diseases raise the possibility of modulation. In recent years, researchers have used dietary interventions, probiotics, and prebiotics in attempts to modulate dysbiosis in cases of allergic respiratory diseases. One birth cohort study found that early and long-term consumption of fresh fruit reduced sensitization to inhaled allergens and asthma symptoms [137]. Another study found that regular dietary fiber intake greatly suppressed allergic reactions and reduced allergic symptoms of nasal rubbing and sneezing [138]. In addition, many researchers have found that taking probiotics and prebiotics can inhibit the production of pro-inflammatory cytokines, reduce the inflammatory response, and relieve the symptoms of allergic diseases [51,53,139]. A meta-analysis of clinical trials showed that probiotics were effective in reducing IgE production and the risk of atopic sensitization [140]. However, the researchers found that uncertain therapeutic targets due to patient heterogeneity made such treatment less effective than they expected [141]. In addition to bacterial modulation, clinical practitioners have sought to block immune pathways targeting respiratory allergic diseases. Dupilumab, a fully human monoclonal antibody which recognizes IL-4Rα and blocks both the IL-4 and IL-13 signals, can reduce the production of airway mucus and improve FEV1 and lung function. In 2019, dupilumab was granted approval for the treatment of uncontrolled asthma with occasional adverse reactions at the injection site. Effective combination of bacterial regulation and immune modulation could be a key to improve the effectiveness of treatment.

It is difficult to precisely identify microorganisms to target for adjustment due to the presence of extensive and various bacteria changes in allergic respiratory diseases. In this review, we considered the key genera associated with allergic respiratory diseases and analyzed the association between these covariant bacteria and immune disorders in cases of allergic diseases. We found that some bacteria commonly changed in the respiratory tract of patients with allergic respiratory diseases; these were linked with allergic immune inflammatory responses, and might be a trigger of allergic respiratory diseases. We also found that bacteria that commonly decreased in the intestinal tract were associated with inhibited allergic immune inflammatory response, and might be controllers of allergic disease. These findings imply that maintaining the stability of intestinal commensal bacteria that can inhibit immune inflammation, and controlling the growth of airway bacteria associated with proinflammatory response, can synergistically prevent allergic immune inflammation, and may provide new strategies for the treatment of allergic respiratory diseases. 

## 6. Conclusions and Future Perspectives

This article provides an extensive overview of current evidence concerning the association between bacterial microbiome and the development of allergic respiratory diseases. In patients with such diseases, among many complicated bacterial changes at different bodily sites, common signatures that might be responsible for allergic respiratory diseases are gradually being identified. A substantial body of evidence suggests that these commonly changed bacteria are associated with diverse immune dysregulation phenomena. However, studies on respiratory bacterial microbiota have been limited in scale, with much variation in sampling methods and sampling sites used, and the evidence supporting the association between immunity and bacteria comes primarily from in vitro cellular studies. This might suggest that the stated association is so far supported by inadequate and unsound evidence, and that large-scale cohort studies and multi-omics analysis, with uniform standard sampling and careful design, together with more mechanistic experiments in animals, are needed to provide stronger support. 

This review suggests new possibilities for the treatment of allergic respiratory diseases. To build on our findings, environmental and other influencing factors affecting these key bacteria changes should be further studied. To this end, we suggest that a combination of modulating the bacterial composition and microenvironment represents the most promising area of potential study.

## Data Availability

Not applicable.

## Supplementary Materials

The following supporting information can be downloaded at: https://www.mdpi.com/article/10.3390/microorganisms10102066/s1, Figure S1: Changes in the bacteria of the upper respiratory and intestinal tracts in allergic respiratory disease; Figure S2: Species changes in nasal and intestinal bacteria in the three diseases. (A). Species changes in the nasal bacteria. (B). Species changes in the intestinal bacteria. Red represents increased abundance, blue represents decreased abundance, yellow represents uncertain changes in abundance and gray represents no significant bacteria change.
