# Supplementary figures and images for: The Changes in Bacterial Microbiome Associated with Immune Disorder in Allergic Respiratory Disease
Source: Microorganisms. 2022 Oct 19;10(10):2066. doi: 10.3390/microorganisms10102066 (PMC9610723; doi:10.3390/microorganisms10102066)

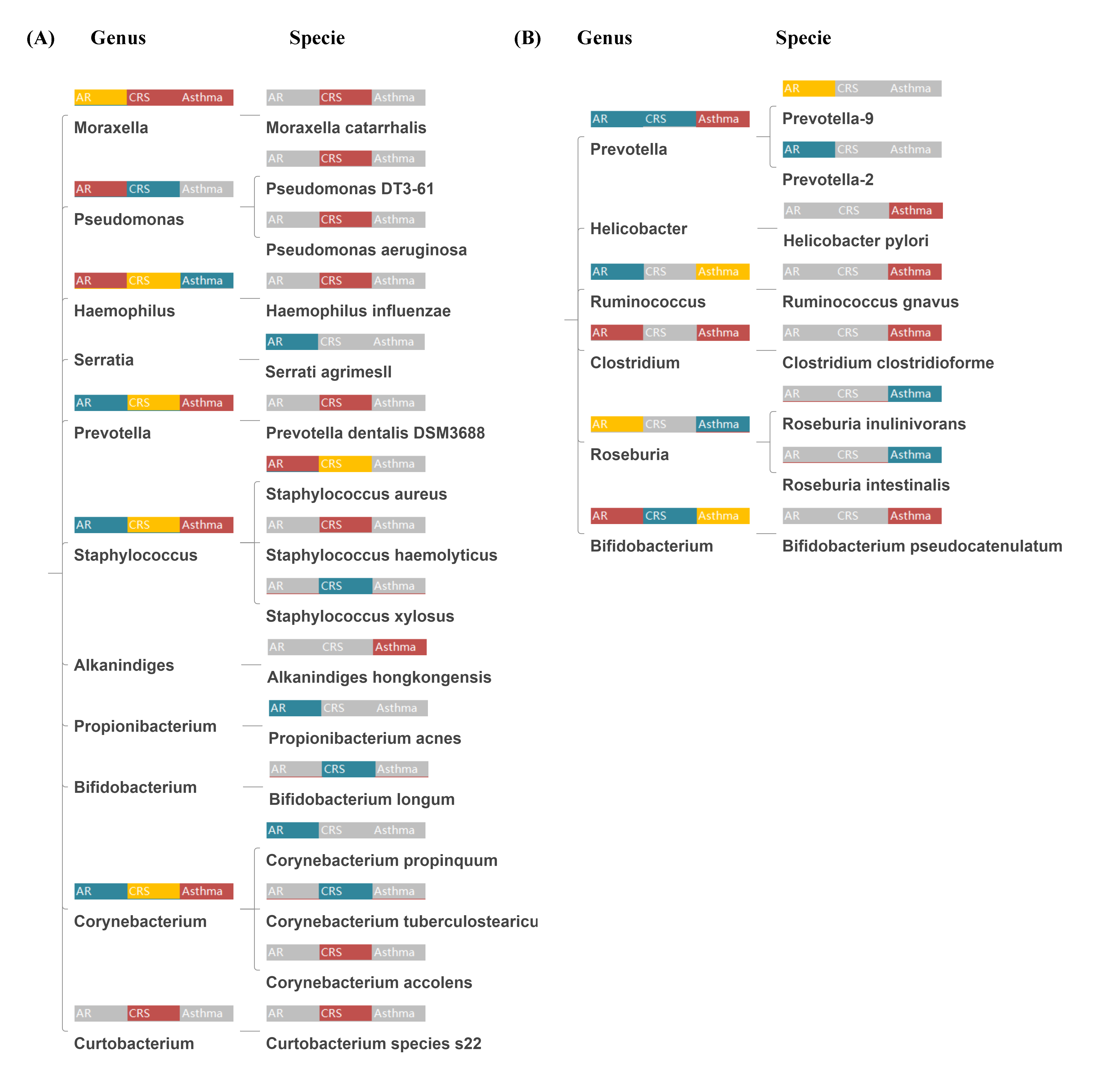

Supplement: Supplementary file 1 [file microorganisms-10-02066-s001.zip › Figure S2.tif]
